# Applicability of a Web-Based, Individualized Exercise Intervention in Patients With Liver Disease, Cystic Fibrosis, Esophageal Cancer, and Psychiatric Disorders: Process Evaluation of 4 Ongoing Clinical Trials

**DOI:** 10.2196/resprot.8607

**Published:** 2018-05-22

**Authors:** Daniel Pfirrmann, Nils Haller, Yvonne Huber, Patrick Jung, Klaus Lieb, Ines Gockel, Krystyna Poplawska, Jörn Markus Schattenberg, Perikles Simon

**Affiliations:** ^1^ Department of Sports Medicine, Disease Prevention and Rehabilitation Institute of Sports Science Johannes Gutenberg University Mainz Germany; ^2^ Department of Psychiatry and Psychotherapy University Medical Center Johannes Gutenberg University Mainz Germany; ^3^ Department of Medicine I University Medical Center Johannes Gutenberg University Mainz Germany; ^4^ Department of Visceral, Transplant, Thoracic and Vascular Surgery University Hospital Leipzig Germany; ^5^ Pediatric Pulmonology Children's Hospital University Medical Center of the Johannes Gutenberg University Mainz Germany

**Keywords:** esophageal cancer, cystic fibrosis, depression, nonalcoholic fatty liver disease, exercise, eHealth

## Abstract

**Background:**

In the primary and secondary prevention of civilization diseases, regular physical activity is recommended in international guidelines to improve disease-related symptoms, delay the progression of the disease, or to enhance postoperative outcomes. In the preoperative context, there has been a paradigm shift in favor of using preconditioning concepts before surgery. Web-based interventions seem an innovative and effective tool for delivering general information, individualized exercise recommendations, and peer support.

**Objective:**

Our first objective was to assess feasibility of our Web-based interventional concept and analyze similarities and differences in a sustained exercise implementation in different diseases. The second objective was to investigate the overall participants’ satisfaction with our Web-based concept.

**Methods:**

A total of 4 clinical trials are still being carried out, including patients with esophageal carcinoma scheduled for oncologic esophagectomy (internet-based perioperative exercise program, iPEP, study), nonalcoholic fatty liver disease (hepatic inflammation and physical performance in patients with nonalcoholic steatohepatitis, HELP, study), depression (exercise for depression, EXDEP, study), and cystic fibrosis (cystic fibrosis online mentoring for microbiome, exercise, and diet, COMMED, study). During the intervention period, the study population had access to the website with disease-specific content and a disease-specific discussion forum. All participants received weekly, individual tailored exercise recommendations from the sports therapist. The main outcome was the using behavior, which was obtained by investigating the log-in rate and duration.

**Results:**

A total of 20 participants (5 from each trial) were analyzed. During the intervention period, a regular contact and a consequent implementation of exercise prescription were easily achieved in all substudies. Across the 4 substudies, there was a significant decrease in log-in rates (*P*<.001) and log-in durations (*P*<.001) over time. A detailed view of the different studies shows a significant decrease in log-in rates and log-in durations in the HELP study (*P*=.004; *P*=.002) and iPEP study (*P*=.02; *P*=.001), whereas the EXDEP study (*P*=.58; *P*=.38) and COMMED study (*P*=.87; *P*=.56) showed no significant change over the 8-week intervention period. There was no significant change in physical activity within all studies (*P*=.31). Only in the HELP study, the physical activity level increased steadily over the period analyzed (*P*=.045). Overall, 17 participants (85%, 17/20) felt secure and were not scared of injury, with no major differences in the subtrials.

**Conclusions:**

The universal use of the Web-based intervention appears to be applicable across the heterogonous collectives of our study patients with regard to age and disease. Although the development of physical activity shows only moderate improvements, flexible communication and tailored support could be easily integrated into patients’ daily routine.

**Trial Registration:**

iPEP study: ClinicalTrials.gov NCT02478996; https://clinicaltrials.gov/ct2/show/NCT02478996 (Archived by WebCite at http://www.webcitation.org/6zL1UmHaW); HELP study: ClinicalTrials.gov NCT02526732; http://www.webcitation.org/6zJjX7d6K (Archived by WebCite at http://www.webcitation.org/6Nch4ldcL); EXDEP study: ClinicalTrials.gov NCT02874833; https://clinicaltrials.gov/ct2/show/NCT02874833 (Archived by WebCite at http://www.webcitation.org/6zJjj7FuA)

## Introduction

There is a worldwide trend toward higher incidence of Barrett cancer [1-3], depression [4], or nonalcoholic fatty liver disease (NAFLD) [5-7], and an alarming increase in overweight and obesity [8]. Being physically active reduces not only the risk for numerous diseases [9] but also stabilizes or slows down disease progression [10-13]. However, chronic conditions require continuous exercise [10,11]. Currently, regular physical activity is recommended in treating chronic diseases according to more elaborated, modern investigations [14,15]. Despite this fact, many patients are unable to perform moderate exercise in the long term [16], mainly due to numerous obstacles in initiating and maintaining an active lifestyle [17]. For instance, studies showed a reduced physical activity level (intensity and amount) in patients with NAFLD compared with healthy controls [18-24]. Changing lifestyle is not easy, especially for this group of patients with sedentary habits [25]. Consequently, regular motivational support from experts to achieve lifestyle changes is recommended [25]. Supervised face-to-face programs with an expert seem also to improve compliance regardless of the type of disease [16,26,27]. However, exercise intensity, duration, and frequency need to be planned carefully for the purpose of enhancing physical health [9]. To provide close support in the long term and in a sustainable manner, which is focused on the flexibility of the patient, the internet seems to be a suitable tool. It has been shown that novel Web-based interventions might be a cost-effective, complementary alternative for a close supervision despite the distance to the real treatment location [28-30]. Outstanding advantages of Web-based interventions are as follows: an easy access regardless of place and time and the anonymous nature [3,29-38]. Therefore, and based on the promising results of previous trials [39-41], we decided to support patients with different diseases (esophageal [Barrett] carcinoma, NAFLD, depression, and cystic fibrosis) with a Web-based, individualized, supervised concept. The common platform for all these patient groups is a key aspect in our concept. We focused on regular feedback and recommended adjusted activity goals instead of self-chosen targets, to improve the fitness as well as disease-specific conditions. In this study, we focus predominantly on the various methodological challenges [42]. Therefore, the following 3 questions will be addressed:

1. Is our Web-based intervention concept feasible in different disorders (assessed by evaluating the log-in rate and log-in duration)?
2. What are the similarities and differences between the diseases in terms of exercise implementation (assessed by evaluating the training time and interruptions)?
3. Are the study participants satisfied with the Web-based concept (assessed by Likert items)?
  Our results may provide planning support for future investigations and study designs.

## Methods

### Design

**Figure 1:**
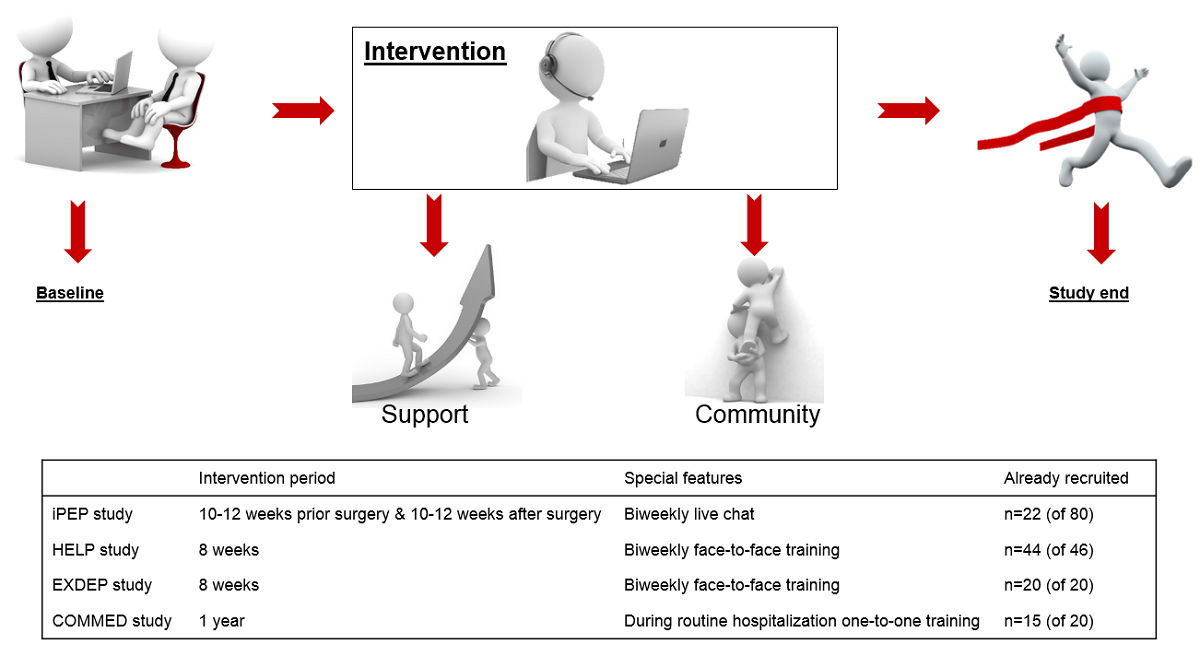
The applied Web-based exercise support concept. HELP: hepatic inflammation and physical performance in patients with NASH; iPEP: internet-based perioperative exercise program; COMMED: cystic fibrosis online mentoring for microbiome, exercise, and diet; EXDEP: exercise for depression.

The webpage went online in 2015. This study consists of 4 substudies that are still ongoing and had separately been approved by the ethics committees. Eligible patients were recruited and screened in 6 University Medical Centers, and all patients provide signed informed consent. In Figure 1, the 4 clinical trials are described.

Due to the diseases studies being different, the primary outcomes and the inclusion and exclusion criteria vary between the substudies. In Table 1 the primary and secondary outcomes of the substudies are summarized. The HELP study (hepatic inflammation and physical performance in patients with nonalcoholic steatohepatitis [NASH]) and the COMMED study (cystic fibrosis online mentoring for microbiome, exercise, and diet) are prospective single-arm trials. The EXDEP study (exercise in depression) and the multicenter iPEP study (internet-based perioperative exercise program) are randomized controlled trials. Patients in the control groups had no access to the webpage (treatment as usual).

Data of patients in the iPEP study were collected at 3 time points. Baseline (t0) was at diagnosis (8-12 weeks before surgery, depending on the date of surgery). The first follow up (t1) was immediately before surgery to show the impact of the exercise program. The final examination (t2) was performed 12 weeks post surgery. In the EXDEP study and HELP study, the patients were tested at study start (t0) and 8 weeks later (t1). In the COMMED study, the study participants were compared with 3 points in time: to study start (t0), 12 weeks later (t1), and after 12 months (t2).

**Table 1:** The primary and secondary outcomes of the 4 clinical trials.

Study	Primary objective	Secondary objective
iPEP^a^	Change of peak oxygen uptake (VO_2peak_)	Gastric conduit failure after esophagectomy; evaluation of postoperative in-hospital stay; quality of life questionnaire QoLQ-C30 with the esophagus-specific module Oesophageal-18
HELP^b^	Change of VO_2peak_	Change of NAS^c^ score at week 0 and week 8
EXDEP^d^	Change of score on the Quick Inventory of Depressive Symptomatology clinician version 16 after 8 weeks compared with baseline; change of score on the Quick Inventory of Depressive Symptomatology self-report version 16 after 8 weeks compared with baseline.	Change of VO_2peak_ after 8 weeks compared with baseline; change of score on the short form-36 after 8 weeks compared with baseline.
COMMED^e^	Changes of fecal and respiratory microbiome, fecal calprotectin, tumor necrosis factor alpha, and VO_2peak_	Changes of FEV1^f^; change of forced vital capacity; change of quality of life

^a^iPEP: internet-based perioperative exercise program.

^b^HELP: hepatic inflammation and physical performance in patients with NASH.

^c^NAS: NAFLD activity score.

^d^EXDEP: exercise for depression.

^e^COMMED: cystic fibrosis online mentoring for microbiome, exercise, and diet.

^f^FEV1: forced expiratory volume in 1 second.

The inclusion criteria in the iPEP study were (1) histologically proven adenocarcinoma of the esophagus or adenocarcinoma of the esophagogastric junction type I according to Siewert’s classification, clinical stages IIB-IIIC (T3/T4 and/or N+; M0) according to Union Internationale Contre le Cancer, 7th Edition; (2) resectable stage according to discussion in the local multidisciplinary tumor board of the participating centers and patient medically fit for multimodality therapy (Eastern Cooperative Oncology Group performance status at least 1 or better, no severe impairment of cardiac, renal, hepatic, endocrine, bone marrow, and cerebral functions); (3) planned abdominal-thoracic esophagectomy with gastric pull-up and intrathoracic or cervical anastomosis; and (4) cognitive ability of the patient to understand the perioperative program and to participate actively.

The exclusion criteria were (1) the presence of a second malignant tumor (unless curatively treated >5 years ago); (2) chemotherapy or radiochemotherapy in patient's history; (3) orthopedic, rheumatologic, cardiovascular, or neurologic (epilepsy, stroke, Parkinson disease, muscle wasting diseases such as amyotrophic lateral sclerosis or multiple sclerosis) contraindications for the sports program; (4) inability to use the internet or no internet access; (5) inability to communicate in German; (6) each active disease that hinders completion of the study; and (7) active alcoholism or illegal drug consumption within the last 6 months before study entry.

The inclusion criteria for the HELP study were (1) histologically proven NASH or fatty liver disease.

The exclusion criteria were (1) bariatric surgery within the last 5 years, (2) body mass index (BMI) <18.5 kg/m^2^ or >45 kg/m^2^, (3) heart attack or stroke within the last 6 months, (4) higher grade coronary artery disease (CADIII-IV), (5) chronic obstructive pulmonary disease (asthma, COPD), (6) renal insufficiency, (7) uncontrolled hypertension or metabolic abnormalities, (8) alcohol consumption >30 g/day (male) and >20 g/day (female), (9) pregnancy, (10) concomitant medication able to cause a secondary NASH (eg, tamoxifen, corticosteroids), (11) concomitant medication able to affect inflammation (eg, tumor necrosis factor antagonists), (12) concomitant anticoagulant medication (eg, phenprocoumon; novel oral anticoagulants, NOAC), (13) other immunological or inflammatory diseases (eg, systemic lupus erythematosus), and (14) musculoskeletal disorders, preventing sport physiological investigations.

The inclusion criteria for the EXDEP study were (1) ability to understand the purpose and risks of the study and provide signed and dated informed consent and authorization to use confidential health information in accordance with national and local subject privacy regulations; (2) aged 18 to 65 years, inclusive, at the time of informed consent; (3) Montreal Cognitive Assessment >26 to exclude cognitive impairment; (4) apart from a clinical diagnosis of major depression or bipolar affective disorder, the subject must be in good health as determined by the Investigator, based on medical history and physical examination; (5) Quick Inventory of Depressive Symptomatology scores >5.

The exclusion criteria were (1) use of antidepressive medications or benzodiazepines at doses that have not been stable for at least 6 weeks before screening; (2) psychotherapy that started less than 8 weeks before screening; (3) any clinically significant psychiatric illness other than major depression or bipolar affective disorder; (4) transient ischemic attack or stroke or any unexplained loss of consciousness within 1 year before screening; (5) any uncontrolled medical or neurological/neurodegenerative condition that, in the opinion of the investigator, might impair treatment compliance and adherence; (6) history of unstable angina, myocardial infarction, chronic heart failure (New York Heart Association Class III or IV), or clinically significant conduction abnormalities (eg, unstable atrial fibrillation) within 1 year before screening; (7) clinically significant 12-lead ECG abnormalities, as determined by the investigator; (8) uncontrolled hypertension defined as: average of 3 systolic blood pressure/diastolic blood pressure readings >65/100 mmHg at screening; (9) history of malignancy or carcinoma, with the following exceptions: (i) subjects with cancers in remission more than 5 years before screening, (ii) subjects with a history of excised or treated basal cell or squamous carcinoma, (iii) subjects with prostate cancer in situ; (10) history of seizure within 2 years before screening; (11) recent history (within 1 year of screening) of alcohol or substance abuse as determined by the investigator, a positive urine drug (due to nonprescription drug) or alcohol test at screening; (12) clinically significant systemic illness or serious infection (eg, pneumonia, septicemia) within 30 days before or during screening; (13) history of HIV, hepatitis C virus, or hepatitis B virus; (14) any other medical conditions (eg, renal disease) that are not stable or controlled, or, which in the opinion of the investigator, could affect the subject's safety or interfere with the study assessments; (15) female subjects who are pregnant or currently breastfeeding; (16) participation in another study; (17) other unspecified reasons that, in the opinion of the investigator or Biogen, make the subject unsuitable for enrollment.

The inclusion criteria for the COMMED study were (1) age >12 years (2) forced expiratory volume in 1 second (FEV1) <90% and >28% of the set point or FEV1 >90% or/and lung clearance index LCI >9.

The exclusion criteria were (1) orthopedic, rheumatologic, cardiovascular, or neurologic contraindications for the sports program; (2) inability to use the internet or no internet access; (3) inability to communicate in German; (4) the absence of consent; (5) further cystic fibrosis–specific criteria: (i) severe pulmonary exacerbation, (ii) FEV1 <27% of the set point (standard value Global Lung Initiative), and (iii) acute infection.

### Cardiopulmonary Exercise Test

Before the start of the study, all eligible study participants performed a stepwise cardiopulmonary exercise test until volitional exhaustion. Each stage of the modified walking protocol lasted for 3 min and intensity was increased by speed and elevation of the treadmill. During the test, heart rate (HR) and respiratory gas analysis were continuously monitored. Furthermore, blood samples from the earlobe were taken at the end of each stage to determine lactate concentration. Subjective degree of exhaustion was measured utilizing the Borg scale (6-20) 30 s before the end of each stage [43].

### Intervention Design

**Figure 2:**
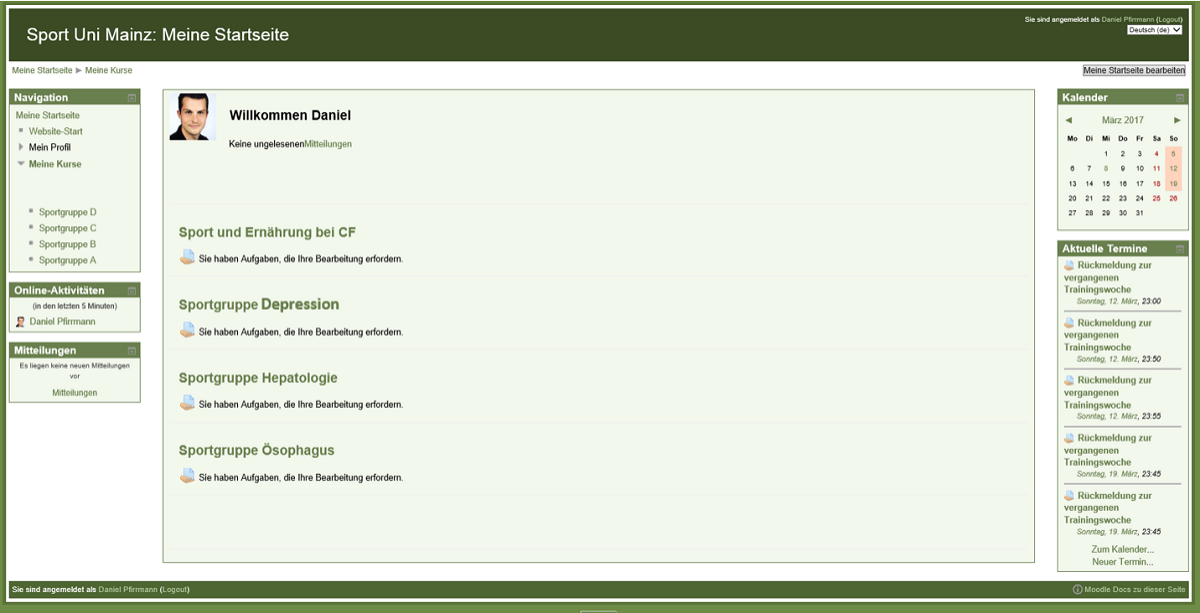
The home screen of the webpage with the divided disease-specific classrooms.

For study design and content structure of the website, experiences from other studies were considered [29,44-46]. Similar to Barak et al (2009), we took the key components of a Web-based intervention into account. We delivered program content, used multimedia aspects, promoted interactive Web-based activities, and provided tailored feedback [37]. Participants were registered on the home page by the administrator and a printed (also online available) instruction manual was provided. Once registered and logged in, users were able to change their profile by editing or deleting information such as username, profile picture, or password. Relevant aspects of data protection were taken into account. The various disease intervention groups obtained access to different parts of the website (Figure 2).

Besides the interaction with a counselor, peer support was considered as a basic principle of our concept. Therefore, each subgroup had its own discussion forum and chat room to improve social support and adherence [46]. Furthermore, nutritional experts or supervising physicians distributed quickly and easily the disease-specific questionnaires and informative documents.

### Exercise Support

**Figure 3:**
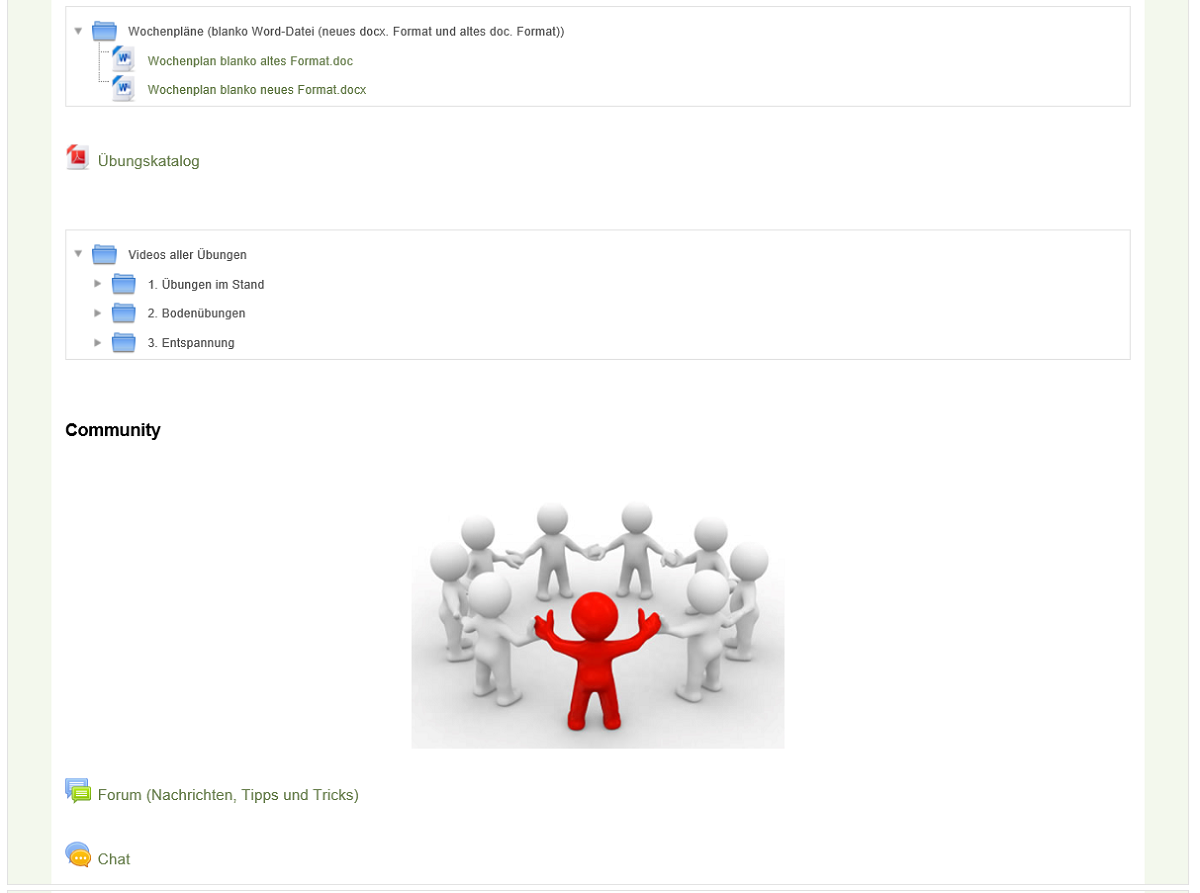
The support area of the home page.

To improve the current condition, sport scientists were responsible for training management and weekly recommendations. Individually tailored exercise plans were sent weekly by an internal email in the secure area of the website. The program consisted of walking or running recommendations, muscle strengthening and stretching exercises, as well as relaxation exercises. An HR monitor (Polar, FT1) was provided to the study participants to monitor the endurance training. The resistance training was carried out in a home-based environment with body-weight exercises and with elastic resistance bands (in different strengths; Pinofit; Pharmazeutische Präparate GmbH, Hamburg) and lasted for approximately 45 min. In addition to an illustrated tutorial, all exercises were additionally deposited as a video file on the home page and could be downloaded or viewed online (Figure 3).

**Figure 4:**
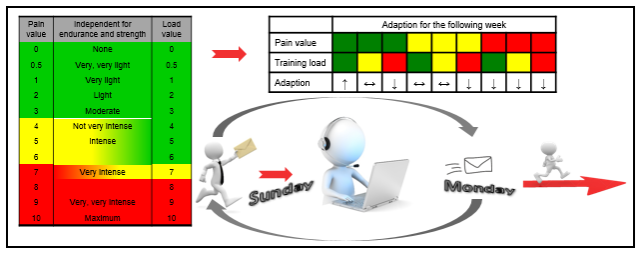
The weekly exercise recommendation structure. Endurance training and strength training were assessed separately.

Participants were encouraged to provide training-related information (eg, average HR, duration, subjective perceived exertion) to the supervisor at the end of each week while filling out a schedule. This information helped the trainers to adapt the training load for the upcoming weeks. Due to the individual feedback of study participants, tailored recommendations focused on the needs, problems, and limitations of each participant were possible. Strength and endurance training were examined separately and allowed specific increase or decrease of training content in terms of duration and/or intensity (Figure 4).

Due to a steady contact with the patients, a missing schedule was noticed quickly by the supervisor sending an email to identify possible problems with the program. Patients were able to contact the trainer at any time by an internal email. In case of questions and feedback, the supervisor answered within 24 hours.

### Measures

The data on using behavior were assessed by evaluating the log-in rate and log-in duration during the intervention period of 8 weeks. The exercise implementation was assessed by evaluating the training time, exercise interruptions, self-chosen alternative exercise programs instead of the weekly recommendations, and adverse events. The user satisfaction with the exercise concept and the webpage was assessed based on a short questionnaire (8 Likert items). Furthermore, the participants graded the concept and were asked if they would continue to use the webpage.

### Analysis

Preliminary descriptive statistics were used to present data on the baseline characteristics of each single trial. Descriptive statistics were also used to show data on utilization and satisfaction with the Web-based exercise concept, as well as exercise adherence and training interruptions and log-in rate and log-in behavior. No data on exercise effects will be presented, due to the heterogeneity of primary outcomes, study collectives, and study designs. Statistical analysis was performed using SPSS (version 22.0, Chicago, IL, USA) and *P* values <.05 were considered significant. Data were not normally distributed and showed heterogeneous variances. Therefore, nonparametric Kruskal-Wallis *H*-tests were carried out to examine trends of physical activity over time among the substudies. The Dunn-Bonferroni test was additionally used as post hoc test to further determine, where exactly the differences between the groups or time points were located.

## Results

### Summary

**Figure 5:**
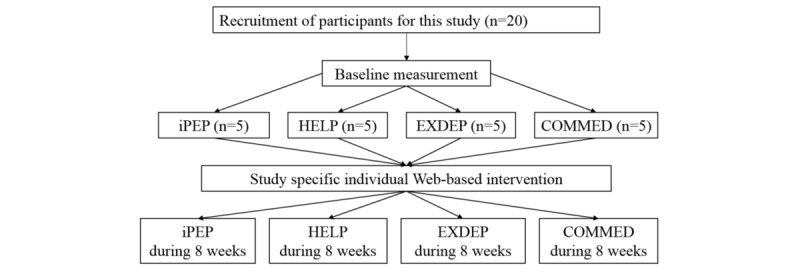
The flowchart of patients’ recruitment. HELP: hepatic inflammation and physical performance in patients with NASH; iPEP: internet-based perioperative exercise program; COMMED: cystic fibrosis online mentoring for microbiome, exercise, and diet; EXDEP: exercise for depression.

All study participants were recruited by physicians in the cooperating centers. Due to different recruiting processes of the single trials and different intervention periods, with regard to the present analysis, we present the first 5 recruited study participants of each study over an 8-week period. As summarized in Table 2, characteristics of the 20 study participants and primary outcome measures at baseline are presented. The patient flow of each trial is shown in Figure 5.

### Participants’ Characteristics

In Table 2, the main baseline characteristics are summarized. The majority of participants were males (n=12) and showed similar distributions with respect to cardiorespiratory fitness. Mean age was 42 years (SD 14.54 years), and mean BMI was 26 kg/m^2^ (SD 3.91 kg/m^2^). Patients in the HELP study tended to be obese (BMI: 30 kg/m^2^; SD: 3.02 kg/m^2^), whereas patients in the COMMED study tended to be underweight (BMI: 22 kg/m^2^; SD: 1.42 kg/m^2^). There was a difference in age across all groups. Patients of the COMMED study were younger (32 years; SD: 7.91 years) due to the congenital disease, whereas Barrett cancer patients in the iPEP study were older (55 years; SD: 5.45 years; *P*<.001). Additionally, there was a significant difference in body weight (*P*<.001) and BMI (*P*<.001) between patients of the COMMED and HELP study. However, a low cardiorespiratory fitness level was common in all substudies compared with sex-specific normative data according to the Heywood classification [47] and the comprehensive investigation by Herdy et al [48].

### Patients’ Acceptance of the Web-Based Concept

#### Are Web-Based Interventions Feasible in Different Disorders?

**Figure 6:**
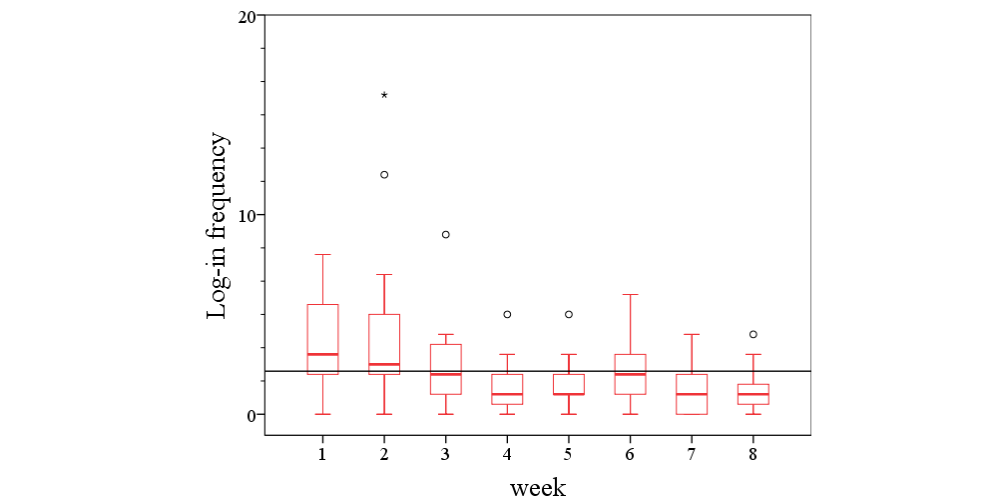
The development of total log-in rate during 8 weeks of intervention.

**Figure 7:**
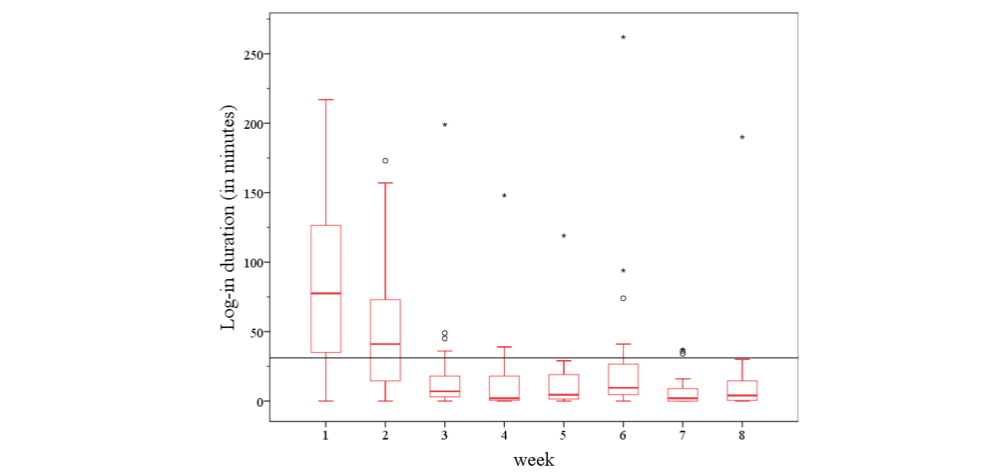
The development of total log-in durations during 8 weeks of intervention.

During the intervention period, a regular contact (at least once a week) and a consequent implementation of exercise prescription were easily achieved in all substudies. The registration process and the detailed explanation took about 1 hour and could be simply integrated in the physical examination at the study start. During the intervention period, there were on average 17 (SD 8.50) log-ins registered across all studies (Table 3). On average, the study participants stayed within 1 log-in for 14 min on the home page. A total of 8 patients (of 20) checked the webpage less than 2 times a week, and 7 (of 20) stayed on average less than 10 min with each stay. Nevertheless, there was a decrease in log-in rates and log-in durations with time. The development of the website utilization is presented in Figures 6 and 7. However, regular communication and the weekly return of the exercise feedback were still realized by email and phone (messenger app) contact.

A detailed view of the different studies shows that all patients of the COMMED study checked the webpage less than 2 times a week and stayed there less than 9 min, whereas the group of the EXDEP study logged-in 2.6 times a week into the webpage and was online for more than 23 min with each stay (Table 3).

**Figure 8:**
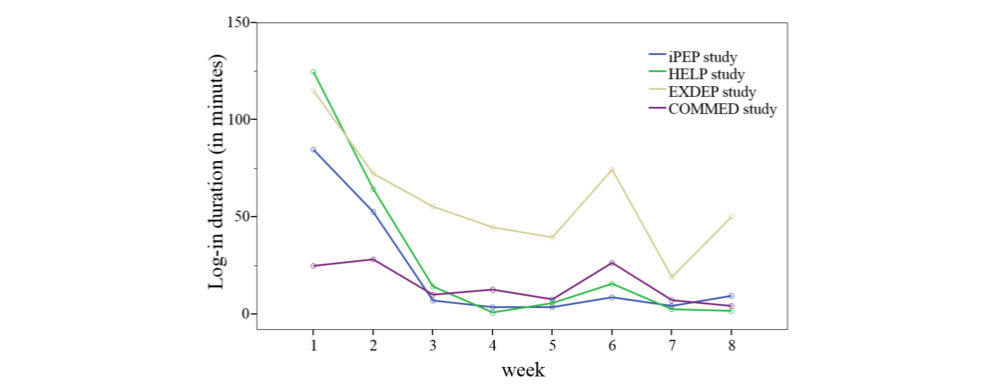
The development of log-in durations during 8 weeks of the intervention for each study. HELP: hepatic inflammation and physical performance in patients with NASH; iPEP: internet-based perioperative exercise program; COMMED: cystic fibrosis online mentoring for microbiome, exercise, and diet; EXDEP: exercise for depression.

**Figure 9:**
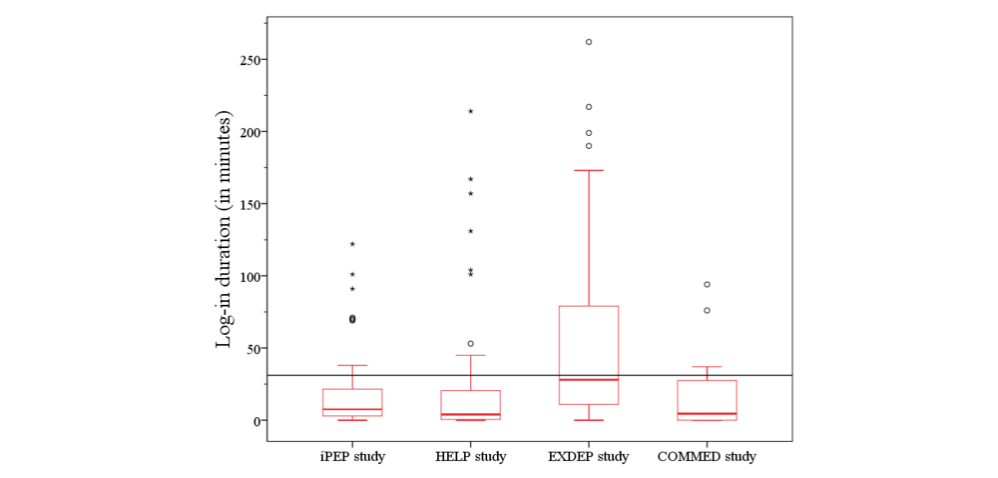
The difference in log-in durations among the substudies. HELP: hepatic inflammation and physical performance in patients with NASH; iPEP: internet-based perioperative exercise program; COMMED: cystic fibrosis online mentoring for microbiome, exercise, and diet; EXDEP: exercise for depression.

During the intervention period, there was a decrease in log-in rates and log-in durations in the HELP study and in the iPEP study, whereas the EXDEP study and COMMED study showed no noteworthy change in the log-in rates and the log-in duration patterns over the 8 weeks of the intervention period. However, there was a higher level in log-in rates and log-in durations in the EXDEP study compared with the COMMED study (Figures 8 and 9). To provide maximum flexibility, the patients were able to contact the study team also by an email or a mobile phone. Of all participants in HELP study, 1 participant called the sports therapist 3 times during the intervention period and sent 3 of the 8 exercise schedules per email. As shown in Figure 8, there is a general lower usage activity in COMMED participants. Of all 5 patients, 3 patients used email contact instead of the webpage for communication with the study team. In total, there were 24 messages sent per email. In the EXDEP study and iPEP study, the communication was realized through the webpage only.

**Table 2:** Baseline patients’ characteristics, demographic data and initial cardiorespiratory results.

Baseline characteristics		iPEP^a^ (N=5)	HELP^b^ (N=5)	EXDEP^c^ (N=5)	COMMED^d^ (N=5)	Total (N=20)
Age, years (SD)		55.2 (5.45)	34.8 (11.38)	49.0 (16.32)	32.4 (7.91)	42.85 (14.54)
**Age groups, n (%)**						
	<30 years	0 (0)	2 (40)	1 (20)	2 (40)	5 (25)
	30-60 years	4 (80)	3 (60)	2 (40)	3 (60)	12 (60)
	>60 years	1 (20)	0 (0)	2 (40)	0 (0)	3 (15)
Women, n (%)		0 (0)	2 (40)	3 (60)	3 (60)	8 (40)
Height, cm (SD)		179.40 (5.96)	174 (12.02)	170.80 (8.39)	169.80 (11.80)	173.50 (10.47)
Weight, kg (SD)		84.6 (6.0)	92.8 (22.7)	75.5 (14.4)	64.8 (7.9)	79.4 (17.0)
BMI^e^, kg/m^2^ (SD)		26.34 (2.36)	30.2 (3.02)	25.84 (3.83)	22.44 (1.42)	26.22 (3.91)
**Spiroergometry**						
	HR^f^ max, bpm (SD)	168 (5.19)	177 (5.47)	162 (23.85)	173 (9.10)	170 (14.26)
	Watt max, W (SD)	132.20 (44.82)	135.20 (44.27)	107.80 (32.01)	108.00 (44.83)	120.80 (42.47)
	VO_2peak_, mL/kg/min (SD)	24.28 (4.37)	28.00 (4.07)	26.88 (8.84)	29.52 (5.02)	27.17 (6.15)
	Borg rating (range 6-20) scale (SD)	17.8 (1.97)	18.6 (1.37)	18.2 (1.62)	17.8 (1.18)	18.1 (1.58)

^a^iPEP: internet-based perioperative exercise program.

^b^HELP: hepatic inflammation and physical performance in patients with NASH.

^c^EXDEP: exercise for depression.

^d^COMMED: cystic fibrosis online mentoring for microbiome, exercise, and diet.

^e^BMI: body mass index.

^f^HR: heart rate.

**Table 3:** Home page usage, log-in frequency, and duration in minutes.

Parameter/variable	iPEP^a^ (N=5)	HELP^b^ (N=5)	EXDEP^c^ (N=5)	COMMED^d^ (N=5)	Total (N=20)
Mean number of log-ins (SD)	21.40 (8.02)	15.20 (6.45)	21.00 (7.71)	11.40 (7.60)	17.25 (8.50)
Average number of log-ins per week (SD)	2.68 (1.00)	1.90 (0.81)	2.63 (0.96)	1.43 (0.95)	2.16 (1.06)
Total duration of log-ins (SD)	173.60 (36.10)	229.20 (111.66)	471.00 (246.07)	121.00 (90.32)	248.70 (195.65)
Average log-in duration (SD)	9.54 (4.16)	17.40 (12.99)	23.41 (13.32)	8.17 (4.38)	14.63 (11.49)

^a^iPEP: internet-based perioperative exercise program.

^b^HELP: hepatic inflammation and physical performance in patients with NASH.

^c^EXDEP: exercise for depression.

^d^COMMED: cystic fibrosis online mentoring for microbiome, exercise, and diet.

#### What Are the Similarities and Differences Between the Diseases in Terms of Exercise Sustained Implementation?

**Figure 10:**
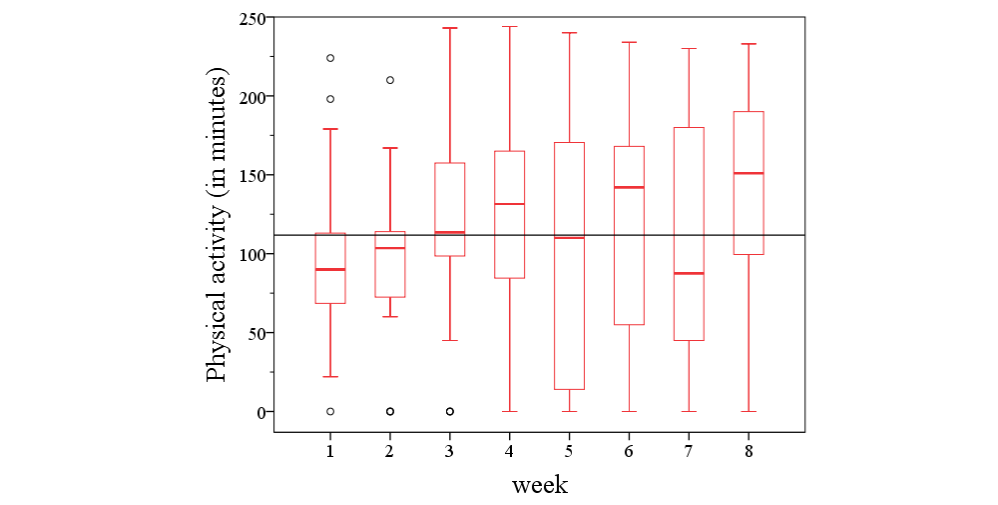
The physical activity development within 8 weeks across all groups.

The training concept was well-tolerated and accepted by the patients in all trials. No severe adverse events occurred during training; however 8 (of 20) patients expressed minor complaints such as blisters or muscle stiffness at least once during the intervention period. In total, 138 exercise recommendations were not performed, due to different reasons. Common reasons for training interruption were because of working reasons (eg, professional development), medical reasons (eg, cold) external conditions (eg, bad weather), or family reasons (eg, illness in the family). The development of the physical activity level over the first 8 weeks period is shown in Figure 10.

**Figure 11:**
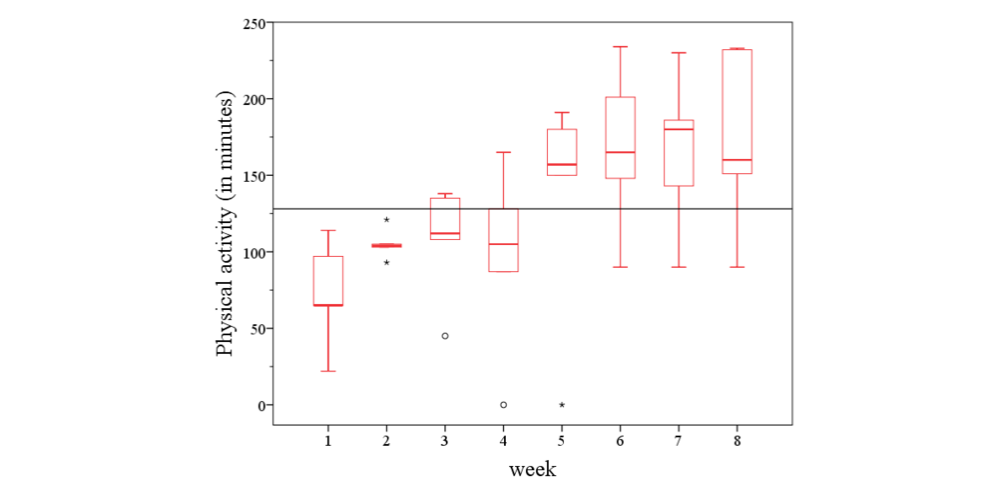
The physical activity development within 8 weeks in the HELP study. HELP: hepatic inflammation and physical performance in patients with NASH.

**Figure 12:**
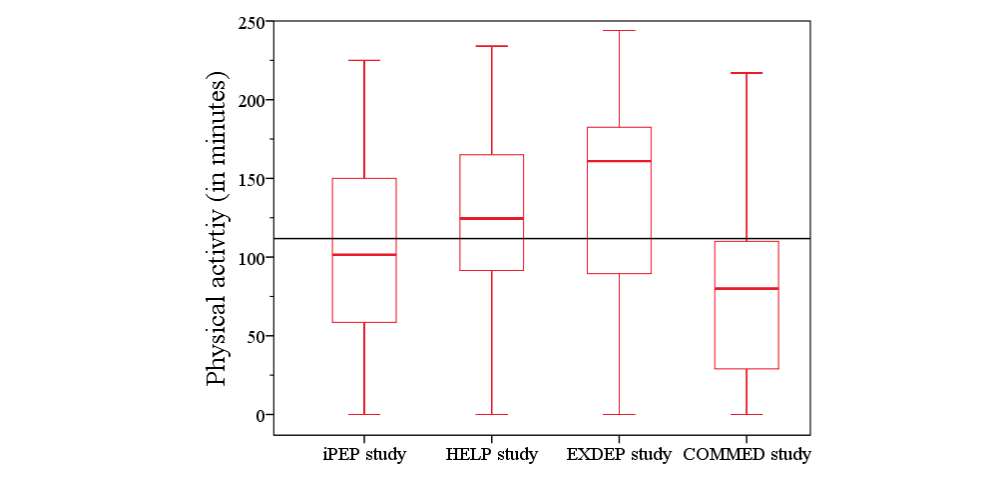
The physical activity level within the 8-week intervention period for each study. HELP: hepatic inflammation and physical performance in patients with NASH; iPEP: internet-based perioperative exercise program; COMMED: cystic fibrosis online mentoring for microbiome, exercise, and diet; EXDEP: exercise for depression.

There was no significant change in physical activity within all studies (χ^2^_7_=8.3, *P*=.311; Figure 10). Only in the HELP study, the physical activity level increased steadily over the period analyzed (χ^2^_7_=14.4, *P*=.045; Figure 11). Statistically significant differences were observed with respect to total physical activity between the substudies. The participants from both the HELP study (*P*=.010) and the EXDEP study (*P*=.001) showed a significant higher activity level compared with the participants of the COMMED study (Figure 12).

However, the development of the physical activity time differed due to different study settings (eg, length of intervention or primary outcome) and the different patient population. Therefore, a comparison of the absolute values between the subgroups was not performed. Especially the 2-phase exercise concept of the iPEP study differed substantially from the other substudies. These patients obtained individual exercise recommendations in the preparation phase for the scheduled surgery. Therefore, an increase in exercise duration was pursued until surgery. However, after surgery and the first weeks of standardized rehabilitation, the exercise concept restarted with reduced advices.

On average, 0.86 (SD 0.75) exercises per week were not performed as instructed (Table 4). We recorded more training interruptions in the COMMED study (mean: 1.13) and the iPEP study (mean: 0.95). Reasons were due to neoadjuvant (radio-/chemo-) therapy in the iPEP cohort and the frequent pulmonary exacerbations of the patients in the COMMED study. Participants in the EXDEP study showed the lowest rate on average with 0.55 interruptions per week. In addition to the recommendations, study participants performed extra workouts or self-chosen alternative exercise programs. In total, 177 alternative sessions were performed. However, 122 of these were completed in the EXDEP study.

#### Are the Study Participants Satisfied With the Web-Based Concept?

A total of 11 questions had to be answered after study end (Table 5). Participants of all study concepts mentioned no fear of getting injured and felt sufficiently supported. Overall, 85% (17/20) felt secure and were not scared of injury. A total of 9 (of 20) would continue to use the webpage and the exercise concept, and 11 (of 20) gave the intervention a grade between 1 and 2 on a grading scale (ranging from 1=very good to 6=unsatisfactory). There were no major differences in the subtrials. A total of 16 (of 20) participants reported that they felt very personally supported despite the distance.

**Table 4:** Physical activity and training interruption.

Variables	iPEP^a^ (N=5)	HELP^b^ (N=5)	EXDEP^c^ (N=5)	COMMED^d^ (N=5)	Total (N=20)
Total physical activity in minutes (SD)	814.20 (286.58)	1024.60 (276.20)	1125.40 (296.22)	639.20 (328.22)	900.85 (349.99)
Average activity per week in minutes (SD)	101.78 (35.82)	128.08 (34.56)	140.68 (37.03)	79.90 (41.03)	112.61 (43.75)
Total training interruption, n (SD)	7.60 (6.42)	6.60 (6.55)	4.40 (3.77)	9.00 (6.04)	6.90 (5.99)
Average number of interruptions per week (SD)	0.95 (0.80)	0.83 (0.82)	0.55 (0.47)	1.13 (0.76)	0.86 (0.75)

^a^iPEP: internet-based perioperative exercise program.

^b^HELP: hepatic inflammation and physical performance in patients with NASH.

^c^EXDEP: exercise for depression.

^d^COMMED: cystic fibrosis online mentoring for microbiome, exercise, and diet.

**Table 5:** Subjective effects and satisfaction.

Question		iPEP^a^ (N=5); n (%)	HELP^b^ (N=5); n (%)	EXDEP^c^ (N=5); n (%)	COMMED^d^ (N=5); n (%)	Total (N=20); n (%)
**How often did you use the webpage (1-10 scale, 1=not at all, 10=several times a day)**						
	1	N/A^e^	N/A	N/A	1 (20)	1 (5)
	2	2 (40)	1 (20)	2 (40)	N/A	5 (25)
	3	N/A	N/A	N/A	N/A	N/A
	4	N/A	2 (40)	2 (40)	2 (40)	6 (30)
	5	1 (20)	2 (40)	N/A	1 (20)	4 (20)
	6	N/A	N/A	N/A	N/A	N/A
	7	N/A	N/A	1 (20)	N/A	1 (5)
	8	N/A	N/A	N/A	N/A	N/A
	9	N/A	N/A	N/A	N/A	N/A
	10	N/A	N/A	N/A	N/A	N/A
	Not stated	2 (40)	N/A	N/A	1 (20)	3 (15)
**How would you rate the training requirements?**						
	1 Very appropriate	3 (60)	5 (100)	3 (60)	2 (40)	13 (65)
	2 Appropriate	N/A	N/A	2 (40)	2 (40)	4 (20)
	3 Undecided	N/A	N/A	N/A	1 (20)	1 (5)
	4 Inadequate	N/A	N/A	N/A	N/A	N/A
	5 Very inadequate	N/A	N/A	N/A	N/A	N/A
	6 Not assessable	N/A	N/A	N/A	N/A	N/A
	Not stated	2 (40)	N/A	N/A	N/A	2 (10)
**How would you rate your fear of injury during training?**						
	1 No fear at all	3 (60)	5 (100)	4 (80)	5 (100)	17 (85)
	2 No fear	N/A	N/A	N/A	N/A	N/A
	3 Undecided	N/A	N/A	N/A	N/A	N/A
	4 Little fear	N/A	N/A	N/A	N/A	N/A
	5 Great fear	N/A	N/A	1 (20)	N/A	1 (5)
	6 Not assessable	N/A	N/A	N/A	N/A	N/A
	Not stated	2 (40)	N/A	N/A	N/A	2 (10)
**How would you rate the injury risk due to unguarded training?**						
	1 No risk at all	3 (60)	5 (100)	4 (80)	3 (60)	15 (75)
	2 No risk	N/A	N/A	1 (20)	2 (40)	3 (15)
	3 Undecided	N/A	N/A	N/A	N/A	N/A
	4 Little risk	N/A	N/A	N/A	N/A	N/A
	5 High risk	N/A	N/A	N/A	N/A	N/A
	6 Not assessable	N/A	N/A	N/A	N/A	N/A
	Not stated	2 (40)	N/A	N/A	N/A	2 (10)
**How would you rate the structure of the webpage?**						
	1 Very simple	2 (40)	3 (60)	3 (60)	2 (40)	10 (50)
	2 Simple	1 (20)	1 (20)	N/A	1 (20)	3 (15)
	3 Undecided	N/A	1 (20)	2 (40)	N/A	3 (15)
	4 Complex	N/A	N/A	N/A	1 (20)	1 (5)
	5 Very complex	N/A	N/A	N/A	N/A	N/A
	6 Not assessable	N/A	N/A	N/A	1 (20)	1 (5)
	Not stated	2 (40)	N/A	N/A	N/A	2 (10)
**How would you rate the communication with the study team?**						
	1 Very personal	3 (60)	5 (100)	5 (100)	3 (60)	16 (80)
	2 Personal	N/A	N/A	N/A	2 (40)	2 (10)
	3 Undecided	N/A	N/A	N/A	N/A	N/A
	4 Impersonal	N/A	N/A	N/A	N/A	N/A
	5 Very impersonal	N/A	N/A	N/A	N/A	N/A
	6 Not assessable	N/A	N/A	N/A	N/A	N/A
	Not stated	2 (40)	N/A	N/A	N/A	2 (10)
**User satisfaction: Did the trainer support meet your expectations?**						
	1 Very satisfied	3 (60)	5 (100)-	5 (100)-	3 (60)	16 (80)
	2 Satisfied	N/A	N/A	N/A	1 (20)	1 (5)
	3 Undecided	N/A	N/A	N/A	N/A	N/A
	4 Dissatisfied	N/A	N/A	N/A	N/A	N/A
	5 Very dissatisfied	N/A	N/A	N/A	N/A	N/A
	6 Not assessable	N/A	N/A	N/A	1 (20)	1 (5)
	Not stated	2 (40)	N/A	N/A	N/A	2 (10)
**Did the feedback of the trainer meet your expectations?**						
	1 Very satisfied	2 (40)	5 (100)	5 (100)	4 (80)	15 (80)
	2 Satisfied	1 (20)	N/A	N/A	N/A	1 (5)
	3 Undecided	N/A	N/A	N/A	N/A	N/A
	4 Dissatisfied	N/A	N/A	N/A	N/A	N/A
	5 Very dissatisfied	N/A	N/A	N/A	N/A	N/A
	6 Not assessable	N/A	N/A	N/A	1 (20)	1 (5)
	Not stated	2 (40)	N/A	N/A	N/A	2 (10)
**Did the structure of the exercise concept meet your expectations?**						
	1 Very satisfied	2 (40)	4 (80)	2 (40)	2 (40)	10 (50)
	2 Satisfied	1 (20)	1 (20)	3 (60)	1 (20)	6 (30)
	3 Undecided	N/A	N/A	N/A	N/A	N/A
	4 Dissatisfied	N/A	N/A	N/A	1 (20)	1 (5)
	5 Very dissatisfied	N/A	N/A	N/A	N/A	N/A
	6 Not assessable	N/A	N/A	N/A	1 (20)	1 (5)
	Not stated	2 (40)	N/A	N/A	N/A	2 (10)
**Give a grade for the concept (1-6 scale, 1=very good, 6=insufficient)**						
	1	2 (40)	1 (20)	1 (20)	N/A	4 (20)
	2	1 (20)	3 (60)	3 (60)	3 (60)	7 (35)
	3	N/A	1 (20)	N/A	N/A	4 (20)
	4	N/A	N/A	N/A	N/A	N/A
	5	N/A	N/A	N/A	1 (20)	1 (5)
	6	N/A	N/A	N/A	N/A	N/A
	Not stated	2 (40)	N/A	1 (20)	1 (20)	4 (20)
**Would you continue to use the webpage? (1-10 scale, 1=unlikely, 10=likely)**						
	1	N/A	N/A	N/A	2 (40)	2 (10)
	2	N/A	N/A	N/A	1 (20)	1 (5)
	3	N/A	N/A	1 (20)	N/A	1 (5)
	4	N/A	1 (20)	1 (20)	1 (20)	3 (15)
	5	1 (20)	N/A	N/A	N/A	1 (5)
	6	2 (40)	N/A	N/A	N/A	2 (10)
	7	N/A	1 (20)	1 (20)	N/A	2 (10)
	8	N/A	1 (20)	N/A	N/A	1 (5)
	9	N/A	1 (20)	N/A	N/A	1 (5)
	10	N/A	1 (20)	1 (20)	1 (20)	3 (15)
	Not stated	2 (40)	N/A	1 (20)	N/A	2 (15)

^a^iPEP: internet-based perioperative exercise program.

^b^HELP: hepatic inflammation and physical performance in patients with NASH.

^c^EXDEP: exercise for depression.

^d^COMMED: cystic fibrosis online mentoring for microbiome, exercise, and diet.

^e^N/A: not applicable.

Table 5 summarizes the subjective satisfaction levels with the Web-based concept. In terms of practicability and structure of the exercise recommendations, patients of the COMMED study stated a lower satisfaction level compared with the participants of the other substudies. This is also reflected in the statement for further usage of the website. Patients of the COMMED study were less interested in a continued use in contrast to patients of the other substudies. The highest interest and satisfaction with the concept could be shown in the HELP and iPEP cohort. At the end of the study, participants mentioned suggestions for improvements. These improvements were dependent on the disease and respective treatments. Thus, participants of the HELP and iPEP study asked for more information and support in nutritional aspects, whereas the younger patient cohort of the COMMED study claimed for app support and a new clear and structured design of the website.

## Discussion

### Principal Findings

This investigation evaluated the feasibility of the Web-based exercise concept for different diseases. The participants of all studies were able to take advantage of the information material and the individual recommendations, irrespective of types of diseases. A quick and easy access to the website enabled a continuous and regular support for the patients. However, a reduced log-in behavior (number and duration) was observed during the time course of the first 8 weeks.

In accordance to Eysenbach et al (2005), less-frequent log-in rates over time are a serious problem of Web-based interventions [42]. According to Couper et al (2007), technical challenges (eg, no access to email, problems accessing or submitting the survey) and problems with the survey (eg, lack of interest in or lack of effectiveness of intervention, no time or bad timing, survey was boring or too long) are the main reasons for noncompletion [49]. Nevertheless, the only common feature of Web-based interventions is the delivery channel [50]. Therefore, the particular role of the website must be evaluated carefully. Regardless of the underlying issue, several features could be frequently used, from computer-generated feedback and general information, such as pamphlets and regular newsletters through to fully tailored feedback and regular contact with peers and the study team (eg, chat or forum function) [51-60]. Besides regular updates, peers on the Web, and general information, the communication to a counselor and the regular monitoring of results by a professional sports therapist seem to be a key reason for compliance and adherence in Web-based settings [34,44,45,61,62]. The log-in rates or durations during the intervention period are influenced by the features offered and are of particular importance in investigations where programs for health education or behavioral change demand regular interaction. In our setting, patients obtained weekly individually tailored exercise recommendations and were encouraged to perform the training in their home environment. The exercise manual for strength training and detailed video files for each exercise could be downloaded and used offline. In accordance to Farvolden et al (2005), this opportunity could explain the high attrition rate over time [53]. Study participants of our investigations got used to the home page and probably preferred viewing the study materials offline. The patients were not obliged to log in several times a week or to be online for special appointments, to complete the study. Furthermore, the patients were able to communicate with the study team via other channels such as apps (WhatsApp), email, or phone contact. The average length of a visit was 14 min and 38 s (SD: 11 min, 29 s), and thus similar to a cognitive behavioral therapy investigation by Farvolden et al (2005) in a collective of patients with panic disorders [53]. A comparable log-in behavior could also be seen in a Web-based physical activity investigation in a cohort of diabetes patients over an 8-week period. The average session duration was 13 min, and the investigators also identified a steep decline in usage of the program toward the end of the study [63]. On the contrary, it is particularly interesting to note that the patients with depressive disorders of our EXDEP study checked the webpage 2.6 times a week and stayed online for more than 23 min. A review article by Vandelanotte et al (2007) summarized the effects of Web-based physical activity interventions in different collectives. Physical activity improvements could be detected in 8 of 15 studies reviewed from 2000 to 2007 [57]. However, essential differences exist between support strategies and the integration of the website content [58-60]. To examine the participants’ perceptions of a physical activity promotion website, Sciamanna et al (2002) provided support in terms of general information materials such as tips for overcoming barriers or instructions for measuring HR and setting activity goals [64]. Furthermore, a printable daily self-monitoring chart of physical activities was available [64]. Similar to our investigation, McKay et al (2001) provided tailored feedback on the basis of the baseline activity level [63]. Study participants received tailored messages to increase the daily activity minutes [63].

In our substudies, regular feedback and tailored recommendations were provided. However, not all study participants followed our instructions. Due to individual situations (eg, well-being vs cancer with neoadjuvant radio/chemotherapy), participants performed alternative exercise programs (eg, relaxation exercise, yoga, or hiking) instead of our recommendation. Therefore, the results in terms of exercise progress during the intervention period should be interpreted with caution. Despite the observed decline in log-in rates and durations toward the end of our substudies, most of the participants (80%, 16/20) were still satisfied with the structure of the concept, which is in accordance with the findings of McKay et al (2001) [63]. It remains unanswered by our present data whether a significant and ongoing decline in log-in behavior, as shown, necessarily means a progressive reduction in exercise adherence.

### Strengths and Limitations

The main strength of this study is the easy access to the website, the opportunity to reach a large group of patients, and the possibility to provide individually tailored exercise support. Furthermore, an immediate reaction on questions, problems, or postings was feasible. Log-in data could be easily collected by tracking the account activity in an objective manner. Finally, the intervention was free of charge and due to minimum investment in human effort, cost effective.

The fact that study participants gave subjective response on their physical activity during a week without an objective evaluation, can be seen as the main disadvantage. The study team had to rely on the participants’ report. Another aspect that needs to be considered, is the website itself. Neither was the forum moderated, nor were regular newsletters distributed, and this possibly led to a reduced log-in activity. Finally, Web-based interventions do reach only selected participants who have access to the internet. Lack of control groups should also be seen as a major limitation. In addition, comparison of the single trials must be interpreted with caution due to the small sample size and the heterogeneous collectives. However, the common platform and same procedure for exercise support might show a general and uniform applicability of the concept used.

### Future Research

Regular updates, intensified monitoring, moderated discussion forums, and additional information materials from other areas of interest, associated with coping with the specific disease, such as nutrition and relaxation, could be added to sustain the website usage in the long run. Furthermore, the integration of app support and modification of the website toward a more intuitive structure as well as the integration of extended functions, such as Web-based forms or data entry, should be realized with regard to subsequent investigations. Finally, investigations with other lifestyle-related diseases, mainly with the metabolic syndrome and its consequences, such as diabetes, obesity, or heart disease, should be focused upon in the future due to its rising incidence.

### Conclusions

The universal use of the Web-based concept appears to be applicable across different diseases and age groups. Although the development of physical activity shows only moderate improvements, flexible communication, timely response to patients’ needs, and tailored support could be easily integrated into patients’ daily routine. However, because of different application habits of the website among the substudies, designing a website that is suitable and sustainable for most users will be a challenging target for future studies. Despite the existing limitations, Web-based approaches can be a helpful supplemental method to bridge the gap between inpatient and outpatient rehabilitation and home treatment for chronically ill patients. An ongoing development in telemedicine makes this kind of intervention with its cost- and time-effectiveness especially interesting for the future.

## Abbreviations

BMI: body mass index
COMMED: cystic fibrosis online mentoring for microbiome, exercise, and diet
EXDEP: exercise for depression
FEV1: forced expiratory volume in 1 second
HELP: hepatic inflammation and physical performance in patients with NASH
HR: heart rate
iPEP: internet-based perioperative exercise program
NAFLD: nonalcoholic fatty liver disease
NASH: nonalcoholic steatohepatitis
